# Allergic Contact Dermatitis to Povidone‐Iodine and Systemic Contact Dermatitis to Chloramphenicol, Used During Intravitreal Injection of Bevacizumab, an Anti‐Vascular Endothelial Growth Factor

**DOI:** 10.1111/cod.14791

**Published:** 2025-03-23

**Authors:** Tommaso Galeotti, Luca Stingeni, Leonardo Bianchi, Marta Tramontana, Katharina Hansel

**Affiliations:** ^1^ Dermatology Section, Department of Medicine and Surgery University of Perugia Perugia Italy

**Keywords:** anti‐vascular endothelial growth factor, bevacizumab, chloramphenicol, intravitreal injection, povidone‐iodine

Diabetic macular edema (DME) is the main cause of visual impairment in patients with diabetic retinopathy (DR) and anti‐vascular endothelial growth factor (anti‐VEGF) therapy is the standard of care for DME [1]. Herein, we report a case of co‐sensitivity to chloramphenicol and povidone‐iodine secondary to intravitreal injection (IVI) of bevacizumab in a patient with bilateral DME.

## 1 Case Report

**FIGURE 1:**
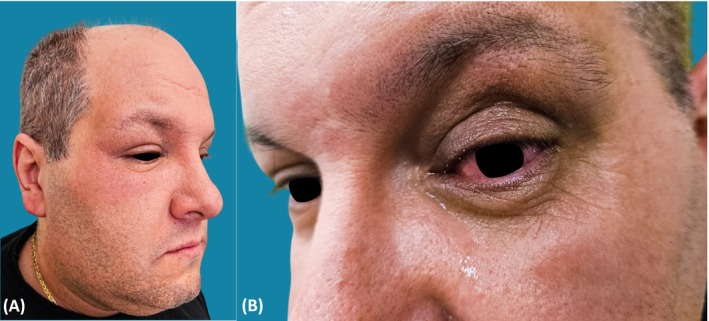
Eczematous dermatitis of the right periocular region after intravitreal injection of bevacizumab (A). Dermatitis relapse of the left periocular region after the subsequent intravitreal injection (B).

A 49‐year‐old man with type‐II diabetes and bilateral DR, complicated by DME of both eyes, was referred to us for an itchy eczematous dermatitis of the periocular region of the right eye, associated with serous conjunctival exudate, subsequently involving the lower two‐thirds of the hemiface (Figure 1A). Skin lesions developed 8 h after the 16th IVI of bevacizumab (25 mg/mL) in the right eye, with no adverse event during the previous 3 years of therapy. Lesions worsened on therapy with betamethasone/chloramphenicol (0.2%/0.5%) ophthalmic ointment and within 2 days, an itchy erythemato‐edematous dermatitis appeared on the antecubital folds, hips, hypogastric region and neck. Topical therapy was discontinued and replaced with gentamicin sulphate (0.1%) ointment and intravenous chlorfenamine maleate (10 mg/1 mL, twice daily), with slight desquamation in 5 days. Without informing us, the patient underwent a new intravitreal injection of bevacizumab in the left eye using the classical povidone‐iodine disinfection, and he developed severe hyperemia, conjunctival exudation (Figure 1B) and eczematous dermatitis localised only on the left face that improved on gentamicin ointment and intravenous chlorfenamine maleate.

In the pre‐injection phase of the intravitreal injection procedure, the patient underwent skin disinfection with a povidone‐iodine cutaneous solution (7.5% in aq.) and topical anaesthesia with lidocaine hydrochloride or oxibuprocaine hydrochloride in both the right and left eyes, while in the post‐injection phase when eczematous dermatitis appeared, he was treated with eye ointment based on chloramphenicol/betamethasone only in the right eye, followed by eye dressings based on hyaluronic acid (0.3%) sodium.

Six weeks after complete resolution, the patient was patch tested with SIDAPA (Società Italiana Dermatologia Allergologica Professionale Ambientale) Baseline Series [2], the Preservatives Integrative Series containing chloramphenicol (5.0% pet.) and povidone‐iodine (10.0% pet.). Bevacizumab (as is, 2.5%), lidocaine hydrochloride (10.0% pet.), oxibuprocaine hydrochloride (0.4% as is) and a fragment of the eye dressing were also patch tested (Table 1). All allergens (except for the eye dressing and oxibuprocaine hydrochloride) were supplied by Smartpractice (Phoenix, AZ, United States). Patch tests were occluded for 2 days (D) with the allergEAZE Test Chambers on Soffix tape (Artsana, Grandate, Italy) and readings were performed on D2, D4 and D7 [3]. A strong positive reaction to povidone‐iodine (++) and an extreme positive reaction to chloramphenicol (+++) were observed at D2 and D4; no new positive reactions were observed at D7. Intradermal tests with bevacizumab and the two topical anaesthetics were negative (Table 1).

**TABLE 1:** Patch and intradermal test results.

Patch and intradermal tests	Results
Patch test	
SIDAPA baseline series	Neg
Topical drugs, excipients, and preservatives integrative series	
Chloramphenicol 5% pet	+++
Povidone‐iodine 10% pet	++
Medicated pad	
Adhesive part as is	Neg
Multi‐layered internal part as is	Neg
External coating part as is	Neg
Bevacizumab solution as is (2.5%)	Neg
Lidocaine hydrochloride (10% pet.)	Neg
Oxibuprocaine hydrochloride eye drops as is (0.4%)	Neg
Intradermal test	
Bevacizumab solution	
0.01% in saline solution	Neg
0.1% in saline solution	Neg
1% in saline solution	Neg
Lidocaine hydrochloride solution	
0.1% in saline solution	Neg
Oxibuprocaine hydrochloride eye drops as is (0.4%)	Neg

Replacing povidone‐iodine in the pre‐injection phase with chlorhexidine aqueous solution (0.1%), and prohibiting ophthalmic products containing chloramphenicol in the post‐injection phase, the patient underwent subsequent intravitreal injections, without recurrence of dermatitis.

## 2 Discussion

Intravitreal injection (IVI) of anti‐VEGF drugs is a standardised therapy currently used for the treatment of DME, other diabetes‐related diseases (wet age‐related macular degeneration, proliferative diabetic retinopathy), retinal vein occlusion, and non‐age‐related macular neovascularization (inflammatory, post‐traumatic, etc.) [1]. According to the current IVI‐guidelines [4], this procedure includes a pre‐injection preparation, an injection phase, and a post‐injection management. The first step includes not only anaesthetic eye drops (eventually with an additional gel or subconjunctival anaesthetic at the site of IVI) [5], but also skin disinfection with povidone‐iodine solution 7.5% to scrub the periocular region. To irrigate the ocular surface and conjunctival sac, eye drops based on povidone‐iodine 5% or chlorhexidine 0.1% aqueous solution, allowed to sit on the eye for at least 30–60 s and then rinsed with sterile physiological solution, are also used [4].

Post‐injection management should involve the use of topical antibiotics, especially in particular conditions (immunodepression, fragile conjunctiva, or intravitreal implant injection) [4], however there is no evidence suggesting that antibiotic use reduces the post‐operative endophthalmitis risk [6].

Chloramphenicol, together with aminoglycoside antibiotics, is a common ophthalmic medication with well‐known sensitising properties due to its widespread use for several infective eye diseases and during ophthalmologic surgical procedures [7]. Our patient developed ACD from povidone‐iodine used twice in the pre‐IVI of the anti‐VEGF procedure. Moreover, chloramphenicol, with a strong positive patch test reaction, that was used only after the first episode of periocular dermatitis with significant worsening, was also very probably responsible for distant lesions on body folds, a form of systemic contact dermatitis due to conjunctival absorption, which has rarely been reported with chloramphenicol [8]. Povidone‐iodine, a well‐known skin irritant, is still widely used as a skin disinfectant in surgery, in the treatment of wounds and leg ulcers, and in ophthalmological medications [9]. In the latter, povidone‐iodine contact allergy mostly derives from the IVI pre‐operative ophthalmological procedure. Povidone‐iodine is the second most frequent allergen after phenylephrine [7], the eye drop mydriatic agent used for ophthalmic examination also in patients undergoing IVI with anti‐VEGF drugs [10].

In conclusion, the increasing IVI of anti‐VEGF drugs, requiring numerous administrations over the years, potentially increases the sensitivity risk to disinfectants and drugs used during the IVI‐procedure. When contact allergy related to IVI is suspected, patch testing performed after careful knowledge of the IVI procedure is necessary for any secondary prevention and to safely continue intravitreal anti‐VEGF therapy.

## Author Contributions

**Tommaso Galeotti:** writing – original draft, data curation, writing – review and editing, methodology. **Luca Stingeni:** conceptualization, investigation, writing – review and editing, methodology, data curation, supervision. **Leonardo Bianchi:** writing – original draft, writing – review and editing. **Marta Tramontana:** writing – original draft, writing – review and editing. **Katharina Hansel:** conceptualization, investigation, methodology, writing – review and editing, data curation, supervision.

## Conflicts of Interest

The authors declare no conflicts of interest.
